# Prevalência de Hipertensão Arterial Sistêmica e Diabetes Mellitus em Indivíduos com COVID-19: Um Estudo Retrospectivo de Óbitos em Pernambuco, Brasil

**DOI:** 10.36660/abc.20200885

**Published:** 2021-08-09

**Authors:** Lucas Gomes Santos, Jussara Almeida de Oliveira Baggio, Thiago Cavalcanti Leal, Francisco A. Costa, Tânia Rita Moreno de Oliveira Fernandes, Regicley Vieira da Silva, Anderson Armstrong, Rodrigo Feliciano Carmo, Carlos Dornels Freire de Souza

**Affiliations:** 1 Universidade Federal de Alagoas Curso de Medicina MaceióAL Brasil Universidade Federal de Alagoas - Curso de Medicina, Maceió, AL - Brasil.; 2 Universidade Federal do Vale do São Francisco Medicina PetrolinaPE Brasil Universidade Federal do Vale do São Francisco – Medicina, Petrolina, PE – Brasil.

**Keywords:** Coronavírus-19, SARS-CoV-19, Pandemia, Hipertensão/complicações, Diabetes Mellitus/complicações, Fatores de Risco/prevenção e controle, Idoso, Prevalência

## Abstract

Hipertensão arterial sistêmica (HAS) e diabetes mellitus (DM) são dois dos principais fatores de risco para a mortalidade por COVID-19.

Descrever a prevalência e o perfil clínico-epidemiológico de óbito por COVID-19 ocorridos em Pernambuco, Brasil, entre 12 de março e 14 de maio de 2020 entre pacientes que possuíam hipertensão arterial sistêmica e/ou diabetes mellitus como doenças prévias.

Estudo observacional transversal. Foram analisadas as seguintes variáveis: município de procedência, sexo, faixa etária, tempo entre o início dos sinais/sintomas e o óbito, sinais/sintomas, tipo de comorbidades e hábitos de vida. Variáveis categóricas foram descritas por meio de frequências e variáveis contínuas por meio de medidas de tendência central e de dispersão. Os testes de Mann-Whitney e Kruskal-Wallis foram utilizados.

Dos 1.276 registros incluídos no estudo, 410 apresentavam HAS e/ou DM. A prevalência de HAS foi 26,5% (n=338) e de DM foi 19,7% (n=252). Dos registros, 158 (12,4%) eram de pacientes que possuíam somente HAS, 72 (5,6%) somente DM e 180 (14,1%) apresentavam HAS e DM. Dos indivíduos com HAS, 53,3% apresentavam DM e 71,4% dos diabéticos apresentam HAS. A mediana (em dias) do tempo entre o início dos sinais/sintomas e o desfecho óbito foi 8,0 (IIQ 9,0), sem diferença significativa entre os grupos de comorbidades (p=0,633), sexo (p=0,364) e faixa etária (p=0,111). Observou-se maior prevalência de DM e HAS na população masculina (DM — 61,3% eram homens e 38,9% mulheres; HAS — 53,2% eram homens e 46,8% mulheres). Os sinais/sintomas mais frequentes foram dispneia (74,1%; n=304), tosse (72,2%; n=296), febre (68,5%; n=281) e saturação de O2<95% (66,1%; n=271). Dos hipertensos, 73,3% (n=100) apresentavam outras comorbidades/fatores de risco associados, e 54,2% (n=39) dos diabéticos apresentavam outras comorbidades/fatores de risco associados. Destacaramse as cardiopatias (19,5%; n=80), obesidade (8,3%; n=34), doença respiratória prévia (7,3%; n=30) e nefropatia (7,8%; n=32). A prevalência de tabagismo foi 8,8% (n=36) e de etilismo alcançou 3,4% (n=14).

O estudo mostrou que a prevalência de HAS foi superior à prevalência de DM nos indivíduos que foram a óbito por COVID-19. Em idosos, a prevalência foi superior à observada em indivíduos não idosos.

## Introdução

Em dezembro de 2019, as entidades sanitárias da província de Hubei, na República popular da China, identificaram e relataram à Organização Mundial da Saúde (OMS) um surto de uma pneumonia com agente etiológico até então desconhecido.^1^ No início de janeiro, o vírus SARS-CoV-2 (*Severe Acute Respiratory Syndrome Coronavírus 2*) foi identificado e a doença foi denominada COVID-19 (*Coronavirus Disease 2019*).^2^

Em 4 de agosto de 2020, a doença já havia infectado um total de 18.316.072 pessoas e causado a morte de 694.715. EUA, Brasil e Índia ocupam as primeiras posições em número de doentes.^3^ No Brasil, o primeiro caso foi confirmado no dia 26 de fevereiro na cidade de São Paulo. Entre essa data e 4 de agosto de 2020, o país somou 2.750.249 infectados e 94.665 óbitos.^4^

Devido ao impacto global causado pela pandemia, existe uma urgência na produção de conhecimento acerca do novo coronavírus. A caracterização das pessoas infectadas é essencial para o planejamento do combate à doença e da retomada econômica. Desde o começo da pandemia, diversos estudos foram publicados com esse intuito, e mostraram que a doença afeta de forma mais grave principalmente pessoas idosas com presença de comorbidades.^5^^,^^6^ A hipertensão arterial sistêmica (HAS) e o diabetes mellitus (DM) são as comorbidades mais frequentes nas pessoas que foram a óbito e sua fisiopatologia parece favorecer o desenvolvimento de quadros mais graves.^7^^–^^9^

Com a doença ainda em expansão em território brasileiro, é importante entender as características das pessoas infectadas no país e também em diferentes estados, devido ao tamanho continental e as diferenças socioeconômicas presentes no Brasil.^10^ O estado de Pernambuco foi particularmente afetado, com registro de 98.833 casos e 6.717 óbitos em 4 de agosto de 2020.^11^

Dessa forma, o presente estudo teve como principal objetivo descrever a prevalência e o perfil clínico-epidemiológico de óbitos por COVID-19 ocorridos em Pernambuco entre 12 de março e 14 de maio de 2020, entre pacientes que possuíam hipertensão arterial sistêmica e/ou diabetes mellitus como doenças prévias.

## Métodos

Trata-se de um estudo observacional transversal, envolvendo todos os óbitos por COVID-19 notificados em Pernambuco entre 12 de março e 14 de maio de 2020, entre pacientes que possuíam HAS e DM como doença de base. No estudo, foram analisadas as seguintes variáveis: sexo, faixa etária, tempo entre o início dos primeiros sintomas e o óbito, sinais/sintomas, a quantidade e o tipo de comorbidades associadas, além da HAS e do DM e hábitos de vida (tabagismo e etilismo). Os dados foram obtidos da página eletrônica de monitoramento da COVID-19 do estado de Pernambuco (https://dados.seplag.pe.gov.br/apps/corona.html) em 15 de maio de 2020. Após a coleta, o banco de dados passou por ajustes das variáveis, que consistiu na adequação dos sinais/sintomas e comorbidades e exclusão de registros inconsistentes.

Para a análise estatística, inicialmente, as variáveis categóricas foram descritas por meio de frequências (absolutas e relativas) e as variáveis contínuas por meio de medidas de tendência central e de dispersão. Para a comparação do tempo de início dos sintomas e o óbito entre os sexos feminino e masculino, utilizou-se o teste de Mann-Whitney, e entre as faixas etárias e grupo de comorbidades, utilizou-se o teste de Kruskal-Wallis com a aplicação posterior de teste *post-hoc*. Considerou-se intervalo de confiança de 95% e significância de 5%.

As análises foram realizadas com o auxílio do *software* SPSS versão 24.0 (IBM Corporation). Por utilizar dados de domínio público, nos quais não é possível a identificação dos indivíduos, este estudo dispensou a aprovação pelo Comitê de Ética em Pesquisa.

## Resultados

Até o dia 14 de maio, constavam 1461 óbitos no banco de dados analisado do estado de Pernambuco. Desses casos, foram excluídos 185 pela baixa qualidade dos dados (ausência e/ou inconsistência entre as variáveis), restando 1.276 óbitos. Desses registros, 338 (26,48%) apresentavam HAS e 252 (19,74%) apresentavam DM como doenças de base: 158 (12,4%) possuíam apenas HAS, 72 (5,6%) apenas DM e 180 (14,1%) HAS+DM. Dos indivíduos com HAS, 53,3% apresentavam DM e 71,4% dos diabéticos apresentavam HAS.

Os óbitos por COVID-19 tendo a HAS como doença de base foram registrados em 56 municípios, com destaque para Recife (n=141), Jaboatão dos Guararapes (n=27), Paulista (n=27) e Olinda (n=17), totalizando 62,72% (n=212) dos óbitos do estado. Já os óbitos por COVID-19 tendo o DM como doença de base foram registrados em 49 municípios, com destaque para Recife (n=104), Jaboatão dos Guararapes (n=21), Olinda (n=13), Cabo de Santo Agostinho (n=12) e Paulista (n=12), na região metropolitana do Recife. Esses quatro municípios somaram 64,28% (n=162) dos óbitos.

**Figura 1:**
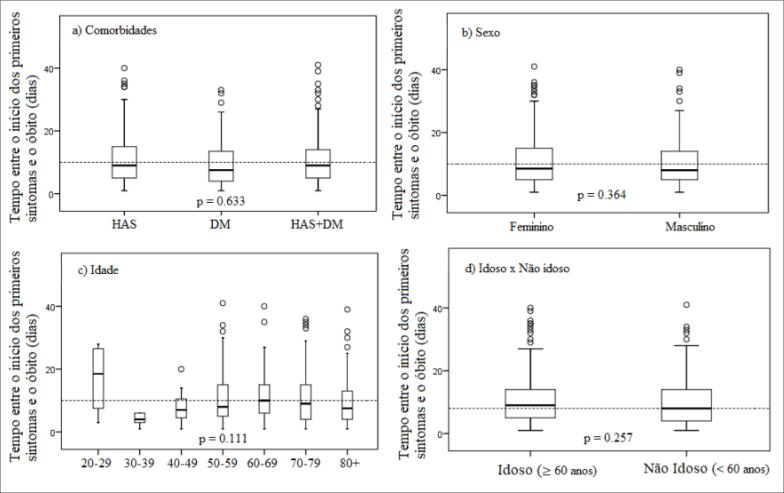
Boxplot do tempo entre o início dos primeiros sintomas e óbito por COVID-19 em indivíduos com hipertensão arterial sistêmica e/ou diabetes mellitus. Pernambuco, Brasil.

A mediana (em dias) do tempo entre o início dos sinais/sintomas e o desfecho óbito foi 8,0 (IIQ 9,0), sem diferença significativa entre os grupos de comorbidades (p=0,633), sexo (p=0,364), faixa etária (p=0,111) e na comparação entre idosos e não idosos (p=0,257) (Figura 1). Quanto ao perfil clínico e epidemiológico, observou-se distribuição homogênea entre os sexos no grupo geral (n=410). No entanto, a análise desagregada mostrou maior prevalência de DM e HAS na população masculina (DM — 61,3% eram homens e 38,9% mulheres; HAS — 53,2% eram homens e 46,8% mulheres). Por outro lado, ao considerar apenas indivíduos com as duas comorbidades, observou-se predomínio de mulheres (53,3%) (Tabela 1).

**Tabela 1:** Caracterização clínica e epidemiológica dos óbitos por COVID-19 entre pacientes que apresentavam hipertensão arterial sistêmica e/ou diabetes mellitus como doença prévia. Pernambuco, Brasil

Variável	HAS (n=158)		DM (n=72)		HAS + DM (n=180)		Total (n=410)	
Sexo	n	%	n	%	n	%	n	%
Feminino	74	46,8	28	38,9	96	53,3	198	48,3
Masculino	84	53,2	44	61,1	84	46,7	212	51,7
**Idade** 1								
20–29	1	0,6	2	2,8	1	0,6	4	1,0
30–39	2	1,3	1	1,4	2	1,1	5	1,2
40–49	9	5,7	4	5,6	10	5,6	23	5,6
50–59	24	15,2	22	30,6	31	17,2	77	18,8
60–69	40	25,3	13	18,1	41	22,8	94	22,9
70–79	38	24,1	22	30,6	55	30,6	115	28,0
80+	44	27,8	8	11,1	40	22,2	92	22,4
**Sinais/sintomas** 2								
Dispneia	111	70,3	54	75,0	139	77,2	304	74,1
Tosse	117	74,1	51	70,8	128	71,1	296	72,2
Febre	110	69,6	46	63,9	125	69,4	281	68,5
Saturação <95%	99	62,7	57	79,2	115	63,9	271	66,1
Dor de Garganta	13	8,2	12	16,7	17	9,4	42	10,2
Diarreia	6	3,8	4	5,6	11	6,1	21	5,1
Vômito	4	2,5	5	6,9	6	3,3	15	3,7
Mialgia	5	3,2	0	0,0	8	4,4	13	3,2
Astenia	6	3,7	1	1,4	4	2,2	11	2,7
**Nº Comorbidades além da comorbidade de base** 3								
Uma comorbidade	58	36,7	33	45,8	0	0,0	91	22,2
Duas comorbidades	68	43,0	28	38,9	82	45,6	178	43,4
Três ou mais	32	20,3	11	15,3	98	54,4	141	34,4
**Comorbidades**								
Cardiopatia	25	15,8	19	26,4	36	20,0	80	19,5
Obesidade	14	8,9	5	6,9	15	8,3	34	8,3
Doença respiratória prévia	16	10,1	3	4,2	11	6,1	30	7,3
Nefropatia	14	8,9	3	4,2	15	8,3	32	7,8
Doença neurológica prévia	13	8,2	6	8,3	8	4,4	27	6,6
Câncer	5	3,2	1	1,4	6	3,3	12	2,9
**Hábitos de vida**								
Tabagismo atual	12	7,6	3	4,2	8	4,4	23	5,6
Tabagismo pregresso	7	4,4	2	2,8	4	2,2	13	3,2
Etilismo atual	5	3,2	2	2,8	4	2,2	11	2,7
Etilismo pregresso	2	1,3	0	0,0	1	0,6	3	0,7

1 Sem registros em indivíduos com idade inferior a 20 anos.

2 Sinais/sintomas e comorbidades com frequência <2,0% foram suprimidos.

3 Comorbidades de base são HAS e DM. HAS: Hipertensão arterial sistêmica; DM: Diabetes mellitus.

Destacou-se ainda a proporção de idosos na população estudada (73,4% possuíam 60 anos ou mais; n=301). Desses, 85,7% (n=258) apresentavam HAS, 59,5% (n=179) apresentavam DM e 45,2% (n=136) apresentavam as duas comorbidades. Os sinais/sintomas mais frequentes foram dispneia (74,1%; n=304), tosse (72,2%; n=296), febre (68,5%; n=281) e saturação de O2<95% (66,1%; n=271) (Tabela 1).

No que concerne às comorbidades/fatores de risco associados, observou-se que 73,3% (n=100) dos hipertensos e 54,2% (n=39) dos diabéticos apresentavam outras comorbidades/fatores de risco associados. No grupo com HAS+DM, esse percentual foi de 54,4% (n=141). Dentre as comorbidades mais comuns, destacaram-se: cardiopatias (19,5%/n=80), obesidade (8,3%; n=34), doença respiratória prévia (7,3%; n=30) e nefropatia (7,8%; n=32). A prevalência de tabagismo (atual ou pregresso) alcançou 8,8% (n=36) e o etilismo (atual ou pregresso) alcançou 3,4% (n=14) (Tabela 1).

## Discussão

A concentração dos óbitos descritos no presente estudo se concentra em municípios de maior porte (Recife e Jaboatão dos Guararapes) e pode relacionar-se ao número de indivíduos expostos e à circulação de pessoas, já que essas são as duas cidades mais populosas do estado. Soma-se a este fato a composição etária da população e da elevada prevalência de doenças crônicas não transmissíveis.^12^ Além disso, a disseminação da COVID-19 em Pernambuco parece seguir o padrão de outros países: a partir de grandes centros urbanos, se dissemina para cidades médias e pequenas.^13^

No que se refere à faixa etária, nota-se que a maior parte dos óbitos ocorreu em pessoas acima de 60 anos, principalmente na faixa etária de 70 a 79 anos, similar ao que tem sido observado em outros países previamente afetados pela pandemia.^7^^,^^8^ O perfil de comorbidades da população brasileira também é um fator a ser levado em consideração. A prevalência de DM é de 9,4% na população geral e se torna ainda mais significante com o aumento da idade, cuja prevalência é de 22,6% na população maior de 60 anos.^14^ Já a prevalência de HAS é de cerca de 24,0%, alcançando 60,9% na população idosa.^15^ Indivíduos com HAS e DM prévio apresentam maior probabilidade de desenvolverem quadros mais graves da COVID-19, por vezes fatais.^16^

Além da idade, o sexo é outra característica relevante. Em revisão realizada por Li et al.,^17^ na China, cerca de 60% dos infectados pelo SARS-CoV-2 eram homens. Resultados semelhantes foram apresentados por Zhou et al.^8^ tanto nos sobreviventes (59% eram homens) quanto nos indivíduos que foram a óbito (70% homens), percentual superior ao observado em nosso estudo. A relação entre o sexo e a COVID-19 ainda não está elucidada, porém o pior desfecho no sexo masculino pode estar relacionado ao maior número de comorbidades presentes nos homens ou uma resposta do sistema imune diferente da observada na população feminina.^17^

O tempo entre o início dos sintomas e o óbito foi menor do que o descrito previamente na literatura (18,5 dias).^6^ No Brasil, a presença de comorbidades cardiovasculares pode reduzir o tempo de vida em até quatro dias.^18^ Entretanto, o resultado do nosso estudo pode estar subestimado, pois é necessário considerar uma possível dificuldade em reconhecer os primeiros sintomas, sobretudo nos indivíduos com condições socioeconômicas precárias e com nível educacional baixo. Além disso, o viés de memória é uma limitação dessa variável.

Em Pernambuco, 43,9% dos óbitos investigados apresentavam HAS e DM simultaneamente. Em investigação realizada na cidade de Nova York envolvendo pacientes hospitalizados, as comorbidades mais frequentes foram HAS (56,6%), obesidade (41,7%) e DM (33,8%), respectivamente.^7^ Essas comorbidades também foram descritas como as mais frequentes em diferentes investigações.^8^^,^^19^^,^^20^ A prevalência dessas doenças tem variado entre os países: na China, por exemplo, a presença dessas doenças é inferior à observada em países como Itália e EUA.^21^

Até o momento, sabe-se que o vírus SARS-CoV-2 liga-se à enzima conversora de angiotensina 2 (ECA-2), diminuindo a atividade desse tipo de receptor e levando a aumento da permeabilidade vascular.^22^ Este receptor tem uma expressão maior nos pulmões e no coração, sendo fundamental para o funcionamento desses sistemas.^23^ Em pacientes com HAS e DM, existe um aumento desse tipo de receptor em comparação com a população saudável, o que pode levar ao desenvolvimento de quadros mais severos da doença.^23^ Além do mais, o SARS-CoV-2 promove lesão endotelial principalmente nos capilares pulmonares, promovendo um estado pró-coagulação, estado vascular inflamatório e de infiltrado celular, o que pode justificar quadros mais graves em pacientes com DM e obesos.^24^^–^^26^

Adicionalmente, os indivíduos com DM parecem apresentar uma resposta ao SARS-CoV-2 com grandes volumes por interferon (IFN) e resposta tardia de Th1/Th17, contribuindo para uma resposta inflamatória mais intensa.^27^ Um recente estudo *in vitro* demonstrou que a concentração de glicose em monócitos estava relacionada a um aumento da replicação viral e produção de citocinas pró-inflamatórias.^28^

O somatório de diferentes comorbidades em um mesmo indivíduo pode resultar em amplificação da resposta inflamatória e favorecer a rápida progressão e/ou agravamento do quadro clínico, reduzindo a sobrevida dos pacientes.^27^^,^^28^ Nessa análise, as comorbidades mais prevalentes associadas ao DM e à HAS foram cardiopatia não especificada e obesidade. Essas comorbidades também foram observadas em estudo conduzido em Nova York, no qual 18,0% dos indivíduos possuíam cardiopatia e 41,7%, obesidade.^5^ Atualmente, a alta prevalência de obesidade tem sido um grave problema de saúde pública na maioria dos países, inclusive no Brasil.

Os hábitos de vida, tais como tabagismo e etilismo, também podem agravar ainda mais esse risco quando relacionado à COVID-19. Indivíduos fumantes, quando infectados, apresentam 3,5 vezes mais chance de desenvolver formas mais agressivas da doença do que não fumantes.^29^ Por conseguinte, a prática aumenta o risco de lesão pulmonar culminando em bronquiolite respiratória crônica, diversos tipos de pneumonia, cânceres e enfisema pulmonar,^30^ que individualmente são fatores de risco para o SARS-CoV-2 e, em conjunto, diminuem a função pulmonar, aumentando a susceptibilidade ao vírus.

Sobre o consumo de bebidas alcoólicas, entende-se que, quando realizado de forma crônica, resulta em aumento das respostas pró-inflamatórias e redução das defesas anti-inflamatórias intermediadas pelas citocinas.^31^ Associado a isso, o sistema imunológico como um todo é prejudicado com a prática do etilismo por reduzir a capacidade de combater agentes infecciosos através da imunidade inata e adaptativa, expondo de forma mais agressiva os contaminados pelo SARS-CoV-2.^31^

Ainda não se conhece os efeitos acumulados das comorbidades no agravamento e mortalidade pela COVID-19. É provável que o somatório de comorbidades possa atuar em conjunto para facilitar tanto a entrada celular do SARS-CoV-2 mediada pela ACE-2 ^26^ nas células quanto favorecer respostas inflamatórias mais agressivas. Estudos sobre esses aspectos são fortemente recomendados.

Mesmo com todos os cuidados metodológicos adotados, este estudo possui limitações: i. A base utilizada é de domínio público e foi construída a partir das fichas de notificação da COVID-19, sem a adequada padronização das variáveis e a ausência de detalhamento das informações (níveis glicêmicos, estágio da obesidade, controle pressórico, dentre outros); ii. Ao longo da pandemia, diferentes formulários de notificação foram sendo implementados, com exclusão e/ou adição de variáveis; e iii. Por se tratar de uma doença nova, sem clareza do rol de sinais/sintomas, é provável que os menos comuns não tenham sido identificados pelos pacientes e registrados, sobretudo no início da pandemia.

## Conclusão

A prevalência de HAS foi superior à prevalência de DM nos indivíduos que foram a óbito por COVID-19. Em idosos, a prevalência foi superior à observada em indivíduos não idosos. Além disso, verificou-se importante acúmulo de comorbidades e fatores de risco. O perfil clínico e epidemiológico foi caracterizado por idosos, sinais/sintomas indicativos de comprometimento respiratório e predomínio de mais de uma comorbidade. Não se observou diferença entre o tempo do início dos primeiros sintomas e o óbito na análise segundo sexo e faixa etária.

Recomendamos estudos que possam estimar o risco de gravidade de acordo com o número e o tipo de comorbidades preexistentes.
