# Supplementary material for: Prevalência de Hipertensão Arterial Sistêmica e Diabetes Mellitus em Indivíduos com COVID-19: Um Estudo Retrospectivo de Óbitos em Pernambuco, Brasil
Source: Arq Bras Cardiol. 2021 Aug 9;117(2):416–22. [Article in Portuguese] doi: 10.36660/abc.20200885 (PMC8395789; doi:10.36660/abc.20200885)
Supplement: Translation

# Translation: Prevalence of Systemic Arterial Hypertension and Diabetes Mellitus in Individuals with COVID-19: A Retrospective Study of Deaths in Pernambuco, Brazil

**Authors:** Lucas Gomes Santos, Jussara Almeida de Oliveira Baggio, Thiago Cavalcanti Leal, Francisco A. Costa, Tânia Rita Moreno de Oliveira Fernandes, Regicley Vieira da Silva, Anderson Armstrong, Rodrigo Feliciano Carmo, Carlos Dornels Freire de Souza

**Affiliations:** 1 MaceióAL Brazil Universidade Federal de Alagoas - Curso de Medicina, Maceió, AL - Brazil.; 2 PetrolinaPE Brazil Universidade Federal do Vale do São Francisco – Medicina, Petrolina, PE – Brazil.

**Keywords:** Coronavirus-19, SARS-CoV-19, Pandemics, Hypertension/complications, Diabetes Mellitus/complications, Risk Factors/prevention and control, Aged, Prevalence

## Introduction

In December 2019, health authorities in Hubei province, in the People’s Republic of China, identified and reported to the World Health Organization (WHO) an outbreak of pneumonia with an unknown etiological agent.^1^ In early January, the SARS-CoV-2 virus (Severe Acute Respiratory Syndrome Coronavirus 2) was identified and the disease was called COVID-19 (Coronavirus Disease 2019).^2^

On August 4, 2020, the disease has infected 18,316,072 people and caused the death of 694,715. The USA, Brazil and India occupy the first positions in number of infected people.^3^ In Brazil, the first case was confirmed on February 26^th^ in the city of São Paulo. Between the first case and August 4, 2020, the country had 2,750,249 people infected and 94,665 deaths.^4^

Due to the global impact caused by the pandemic, there is an urgent need to produce knowledge about the new coronavirus. The characterization of infected people is essential for tackling the disease and for economic recovery. Since the beginning of the pandemic, several studies have been published for this purpose, and have shown that the disease affects more severely elderly people with comorbidities.^5^^,^^6^ Systemic arterial hypertension (SAH) and diabetes mellitus (DM) are the most frequent comorbidities in people who have died, and their pathophysiology seems to favor the development of more severe conditions.^7^^–^^9^

COVID-19 is still expanding in Brazil and, because of that, it is important to understand the characteristics of infected people in the country and also in different states, due to Brazil’s continental size and socioeconomic differences.^10^ The state of Pernambuco was particularly affected, with a record of 98,833 cases and 6,717 deaths by August 4, 2020.^11^

This study aimed to describe the prevalence and the clinical and epidemiological profile of deaths from COVID-19 in Pernambuco between March 12 and May 14, 2020, among people that had SAH and/or DM as previous diseases.

## Methods

This is a cross-sectional observational study involving all deaths from COVID-19 reported in Pernambuco, between March 12 and May 14, 2020, of people that had SAH and DM as previous diseases. We analyzed the following variables: sex, age group, time between the onset of the first symptoms and death, signs/symptoms, the number and type of associated comorbidities, in addition to SAH and DM and lifestyle habits (smoking and alcoholism). The data were obtained from the monitoring page of COVID-19 in the state of Pernambuco (https://dados.seplag.pe.gov.br/apps/corona.html) on May 15, 2020. After data collection, we made some adjustments in the database, which consisted of adjustment of signs/symptoms and comorbidities and exclusion of inconsistent records. For statistical analysis, categorical variables were initially described by frequencies (absolute and relative) and continuous variables by measures of central tendency and dispersion. The Mann-Whitney test was used to compare the time between symptom onset and death between females and males and the Kruskal-Wallis test was used to compare the age groups and comorbidities with the subsequent application of a post-hoc test. We adopted a confidence interval of 95% and a significance level of 5%. The analyses were performed with SPSS software version 24.0 (IBM Corporation). This study used public domain data, in which it is not possible to identify individuals. For this reason, approval by the Research Ethics Committee was not necessary.

## Results

Until May 14, there were 1,461 deaths in the state of Pernambuco according to the database analyzed. We excluded 185 cases due to low-quality data (absence and/or inconsistency among the variables), resulting in 1,276 deaths. According to the records, 338 (26.48%) had SAH and 252 (19.74%) had DM as previous diseases: 158 (12.4%) had only SAH, 72 (5.6%) only DM and 180 (14.1%) had SAH + DM. 53.3% of the individuals with SAH had DM and 71.4% of diabetics had SAH.

SAH people died from COVID-19 in 56 cities, including Recife (n=141), Jaboatão dos Guararapes (n=27), Paulista (n=27) and Olinda (n=17), totaling 62.72% (n=212) of deaths in the state. People with DM died from COVID-19 in 49 cities, including Recife (n=104), Jaboatão dos Guararapes (n=21), Olinda (n=13), Cabo de Santo Agostinho (n=12) and Paulista (n=12), in the metropolitan region of Recife. These four cities accounted for 64.28% (n=162) of all deaths.

The median (in days) time between the onset of signs/symptoms and death was 8.0 (IIQ 9.0), with no significant difference between groups of comorbidities (p=0.633), sex (p=0.364), age group (p=0.111) and in the comparison between elderly and non-elderly individuals (p=0.257) (Figure 1). The clinical epidemiological profile showed a homogeneous distribution between sexes in the general group (n=410). However, the disaggregated analysis showed a higher prevalence of DM and SAH in the male population (DM — 61.3% were men and 38.9% women; SAH — 53.2% were men and 46.8%, women). On the other hand, considering only individuals with both comorbidities, there was a predominance of women (53.3%) (Table 1).

**Figure 1:** Boxplot from the onset of first symptoms and death of individuals with COVID-19 and systemic arterial hypertension and/or diabetes in Pernambuco, Brazil. SAH: Systemic arterial hypertension; DM: Diabetes mellitus.

**Table 1:** Clinical epidemiological characterization of deaths from COVID-19 with systemic arterial hypertension and diabetes mellitus as previous diseases, in Pernambuco, Brazil.

Variable	SAH (n=158)		DM (n=72)		SAH + DM (n=180)		Total (n= 410)	
Sex	n	%	n	%	n	%	n	%
Female	74	46,8	28	38.9	96	53.3	198	48.3
Male	84	53.2	44	61.1	84	46.7	212	51.7
**Age** 1								
20–29	1	0.6	2	2.8	1	0.6	4	1.0
30–39	2	1.3	1	1.4	2	1.1	5	1.2
40–49	9	5.7	4	5.6	10	5.6	23	5.6
50–59	24	15.2	22	30.6	31	17.2	77	18.8
60–69	40	25.3	13	18.1	41	22.8	94	22.9
70–79	38	24.1	22	30.6	55	30.6	115	28.0
80+	44	27.8	8	11.1	40	22.2	92	22.4
**Signs/symptoms** 2								
Dyspnea	111	70.3	54	75.0	139	77.2	304	74.1
Cough	117	74.1	51	70.8	128	71.1	296	72.2
Fever	110	69.6	46	63.9	125	69.4	281	68.5
O2 saturation <95%	99	62.7	57	79.2	115	63.9	271	66.1
Sore throat	13	8.2	12	16.7	17	9.4	42	10.2
Diarrhea	6	3.8	4	5.6	11	6.1	21	5.1
Vomit	4	2.5	5	6.9	6	3.3	15	3.7
Myalgia	5	3.2	0	0.0	8	4.4	13	3.2
Asthenia	6	3.7	1	1.4	4	2.2	11	2.7
**Number of Comorbidities behind SAH and DM**								
One comorbidity	58	36.7	33	45.8	0	0.0	91	22.2
Two comorbidities	68	43.0	28	38.9	82	45.6	178	43.4
Three or more	32	20.3	11	15.3	98	54.4	141	34.4
**Comorbidities**								
Cardiopathy	25	15.8	19	26.4	36	20.0	80	19.5
Obesity	14	8.9	5	6.9	15	8.3	34	8.3
Previous respiratory disease	16	10.1	3	4.2	11	6.1	30	7.3
Nephropathy	14	8.9	3	4.2	15	8.3	32	7.8
Previous neurological disease	13	8.2	6	8.3	8	4.4	27	6.6
Cancer	5	3.2	1	1.4	6	3.3	12	2.9
**Lifestyle Habits**								
Current smoking	12	7.6	3	4.2	8	4.4	23	5.6
Previous smoking	7	4.4	2	2.8	4	2.2	13	3.2
Current alcohol consumption	5	3.2	2	2.8	4	2.2	11	2.7
Previous alcohol consumption	2	1.3	0	0.0	1	0.6	3	0.7

1 No records of individuals under 20.

2 Signs/symptoms and comorbidities with frequency <2.0% were suppressed. SAH: Systemic arterial hypertension; DM: Diabetes mellitus.

The proportion of elderly people in the studied population also stood out (73.4% were 60 years old or older; n=301). Of these, 85.7% (n=258) had SAH, 59.5% (n=179) DM and 45.2% (n=136) had the two comorbidities. The most frequent signs/symptoms were dyspnea (74.1%; n=304), cough (72.2%; n=296), fever (68.5%; n=281) and O_2_ saturation <95% (66.1%; n=271) (Table 1).

Regarding comorbidities/associated risk factors, it was observed that 73.3% (n=100) of hypertensive patients and 54.2% (n=39) of diabetics had other comorbidities/associated risk factors. In the group with SAH + DM, this percentage was 54.4% (n=141). The most frequent comorbidities were: heart disease (19.5%/n=80), obesity (8.3%; n=34), previous respiratory disease (7.3%; n=30) and nephropathy (7.8%; n=32). The prevalence of smoking (current or previous) was 8.8% (n=36) and alcoholism (current or previous), 3.4% (n=14) (Table 1).

## Discussion

The majority of deaths described in the present study is concentrated in larger cities (Recife and Jaboatão dos Guararapes) and may be related to the number of individuals exposed to SARS-CoV-2 virus and the high number of people moving around, since these are the two most populous cities in the state. Added to this, the deaths can also be explained by age composition and the high prevalence of chronic non-communicable diseases.^12^ The dissemination of COVID-19 in Pernambuco seems to follow the pattern of other countries: from large urban centers, it spreads to medium and small cities.^13^

The deaths occurred mainly among people older than 60, especially from 70 to 79, which is similar to other countries previously affected by the pandemic.^7^^,^^8^ The profile of comorbidities in the Brazilian population is also a factor to be taken into account. The prevalence of DM is 9.4% in the general population and is even more significant with increasing age, whose prevalence is 22.6% in the population older than 60.^14^ The prevalence of SAH is around 24.0%, and 60.9% in the elderly population.^15^ Individuals with SAH and DM are more likely to develop severe cases of COVID-19 and sometimes it is fatal.^16^

In addition to age, sex is another relevant feature. In a review by Li et al.,^17^ in China, about 60% of people infected with SARS-CoV-2 were men. Similar results were presented by Zhou et al.,^8^ both in survivors (59% were men) and in individuals who died (70% men), a higher percentage of men. The relationship between sex and COVID-19 is still unclear, but the worst outcome in males may be related to the greater number of comorbidities present in men or an immune system response different from that observed in the female population.^17^

The time between the onset of symptoms and death was shorter than that previously described in the literature (18.5 days).^6^ In Brazil, the presence of cardiovascular comorbidities can reduce lifespan by up to four days.^18^ However, the findings of our study may be underestimated, as it is necessary to consider a potential difficulty in recognizing the first symptoms, especially in individuals with precarious socioeconomic conditions and low educational level. In addition, memory bias is a limitation of this variable.

In Pernambuco, 43.9% of the deceased individuals investigated had SAH and DM simultaneously. In a study carried out in New York City involving hospitalized patients, the most frequent comorbidities were SAH (56.6%), obesity (41.7%) and DM (33.8%), respectively.^7^ These comorbidities have also been described as the most frequent ones in different investigations.^8^^,^^19^^,^^20^ The prevalence of these diseases varied between countries: in China, for example, the presence of these diseases is lower than that observed in countries like Italy and the USA.^21^

So far, it is known that the SARS-CoV-2 virus binds to the angiotensin-converting enzyme 2 (ACE-2), decreasing the activity of this type of receptor, increasing vascular permeability.^22^ This receptor has a greater expression in the lungs and heart, being fundamental for the functioning of these systems.^23^ In patients with SAH and DM, there is an increase in this type of receptor compared to the healthy population, which may lead to the development of more severe diseases.^23^ Furthermore, SARS-CoV-2 promotes endothelial damage mainly in the pulmonary capillaries, promoting a pro-coagulation state, inflammatory vascular state and cell infiltrate, which may justify more severe conditions in patients with DM and obese people.^24^^–^^26^

Additionally, individuals with DM appear to have a response to SARS-CoV-2 with large volumes of interferon (IFN) and a late Th1/Th17 response contributing to a more intense inflammatory response.^27^ A recent in vitro study demonstrated that the concentration of glucose in monocytes was related to increased viral replication and production of pro-inflammatory cytokines.^28^

The sum of different comorbidities in the same individual may result in amplification of inflammatory response and favor the rapid progression and/or worsening of the clinical condition, reducing patient survival.^27^^,^^28^ In this analysis, the most prevalent comorbidities associated with DM and SAH were unspecified heart disease and obesity. These comorbidities were also observed in the New York study, in which 18.0% of the individuals had heart disease and 41.7% were obese.^5^ Currently, the high prevalence of obesity has been a serious public health problem in most countries, including Brazil.

Lifestyle habits, such as smoking and excessive alcohol consumption, may also aggravate COVID-19. When infected, smokers are 3.5 times more likely to develop aggressive forms of the disease than non-smokers.^29^ Therefore, smoking increases the risk of lung injury culminating in chronic respiratory bronchiolitis, various types of pneumonia, cancer and pulmonary emphysema,^30^ which, individually, are risk factors for SARS-CoV-2 and, together, decrease lung function, increasing virus susceptibility.

Chronic consumption of alcoholic beverages results in increased pro-inflammatory response and reduced anti-inflammatory defenses mediated by cytokines.^31^ Associated to this, the immune system is impaired because it reduces the ability to fight against infectious agents through innate and adaptive immunity, exposing those infected by SARS-CoV-2 to a more aggressive forms of the disease.^31^

The cumulative effects of comorbidities on aggravation and mortality by COVID-19 is unknown. It is possible that the sum of comorbidities may act together to facilitate both the cellular entry of SARS-CoV-2 mediated by ACE-2^26^ into the cells and favor more aggressive inflammatory responses. Studies on this aspect are strongly recommended.

Even with all the methodological precautions adopted, this study has limitations: i. The database used is in the public domain and was built from the COVID-19 notification forms, without adequate standardization of variables and lack of detailed information (glycemic levels, obesity stage, pressure control, among others); ii. Throughout the pandemic, different notification forms were implemented, excluding and/or adding variables; and iii. As it is a new disease, without a clear list of signs/symptoms, it is likely that the less common ones were not identified by patients and registered, especially at the beginning of the pandemic.

## Conclusion

The prevalence of SAH was higher than the prevalence of DM in individuals who died from COVID-19. In the elderly, the prevalence was higher than that observed in non-elderly individuals. In addition, there was an important accumulation of comorbidities and risk factors. The clinical epidemiological profile was characterized by elderly people, signs/symptoms indicative of respiratory impairment and predominance of more than one comorbidity. There was no difference between the time of onset of the first symptoms and death in the analysis according to sex and age group.

We recommend studies that can estimate the risk of severity according to the number and type of pre-existing comorbidities.
